# Antimicrobial Efficacy of GS-2 on Reusable Food Packaging Materials for Specialty Crops

**DOI:** 10.3390/foods13213490

**Published:** 2024-10-31

**Authors:** Catherine W. Y. Wong, Thomas Burton, Julio Carrera Montoya, Nupoor Birje, Xinyi Zhou, Joelle K. Salazar, Jason M. Mackenzie, Thomas F. Rau, Max Teplitski, Wei Zhang

**Affiliations:** 1 Institute for Food Safety and Health, Department of Food Science and Nutrition, Illinois Institute of Technology, 6502 S Archer Rd., Bedford Park, IL 60501, USA; cwong21@iit.edu (C.W.Y.W.);; 2 Department of Microbiology & Immunology, The Peter Doherty Institute for Infection & Immunity, University of Melbourne, 792 Elizabeth Street, Melbourne, VIC 3000, Australia; tom.burton@unimelb.edu.au (T.B.); jason.mackenzie@unimelb.edu.au (J.M.M.); 3 Division of Food Processing Science and Technology, U.S. Food and Drug Administration, 6502 S Archer Rd., Bedford Park, IL 60501, USA; joelle.salazar@fda.hhs.gov; 4 Wintermute Biomedical Ltd., Corvallis, MT 59828, USA; tom@wintermutebiomedical.com; 5 International Food Produce Association, 1901 Pennsylvania Ave. NW Suite 1100, Washington, DC 20006, USA; mteplitski@freshproduce.com

**Keywords:** *Aspergillus niger*, *Escherichia coli* O157:H7, *Listeria monocytogenes*, *Salmonella enterica*, murine norovirus, acrylonitrile butadiene styrene, cardboard, high-density polyethylene, polypropylene, food safety, fresh produce, storage

## Abstract

The European Union (EU) regulations mandate 10% of all food packaging to be reusable by 2030. United States (U.S.) exporters of specialty crops face new challenges in ensuring microbiological food safety using reusable packaging. A novel antimicrobial formulation consisting of ammonium carboxylate salt of capric acid and L-arginine (GS-2) was recently developed as a spray coating chemical for food packaging materials. In this study, we evaluated the antimicrobial efficacy of GS-2 against microbial strains representing three foodborne bacterial pathogens (*Escherichia coli* O157:H7, *Listeria monocytogenes*, *Salmonella enterica*), one fungal spoilage organism (*Aspergillus niger*), and one surrogate viral pathogen (murine norovirus) on three reusable plastic materials (acrylonitrile butadiene styrene, high-density polyethylene, and polypropylene) and one cardboard packaging material, respectively. Different chemical concentrations, exposure times, and storage conditions were individually evaluated for the relative antimicrobial efficacies of GS-2 against these microorganisms. Our results showed that GS-2 was highly effective for inactivating bacterial pathogens on both plastic and cardboard surfaces. For instance, 3% GS-2 achieved a >5 log CFU/in^2^ reduction in *E*. *coli* O157:H7, *L. monocytogenes*, and *S. enterica* on tested plastic surfaces at an exposure time of 60 min. However, its efficacy against *A. niger* and murine norovirus was less optimal, resulting in a ≤1 log CFU/in^2^ reduction on all tested surfaces. Based on our study, GS-2 demonstrated a strong potential as an antibacterial coating reagent for reusable food packaging materials to minimize pathogen contamination and ensure the safety of the specialty crops.

## 1. Introduction

In response to the growing environmental concerns and demand for sustainable agricultural practices, the European Union (EU) has implemented stringent regulations aimed at reducing single-use packaging materials, particularly single-use plastics. The foundation for these EU regulations was established with the “EU Packaging and Packaging Waste Directive” of 1994, which delineated the types of packaging expected to be reused, including primary, secondary, and tertiary packaging [1]. This directive also provided an operational definition of “reuse” and mandated that recycled or reused packaging comply with hygiene, health, and consumer safety provisions. Building on this framework, the European Commission’s “A European Strategy for Plastics in a Circular Economy” set a goal to ensure that at least 10% of packaging be reusable rather than merely recyclable by 2030 [2]. This regulation further prioritizes food safety considerations for reusable packaging in contact with food, which adds an additional layer of complexity for U.S. exporters who must now address both environmental and food safety challenges [3].

The European Circular Economy regulation is expected to have a significant impact on U.S. exporters of fresh produce, particularly for tree nuts, fresh citrus, and sweet potatoes destined for the EU market [2,4]. When choosing reusable packaging for fresh produce, an important consideration is microbial food safety. In the five-year time period from 2019 to 2024, there have been 31 fresh produce-associated foodborne illness outbreaks in the U.S. alone due to three bacterial pathogens: *Escherichia coli* (*E. coli*), *Listeria monocytogenes* (*L. monocytogenes*), and *Salmonella enterica* (*S. enterica*) [5]. The resulting cost from foodborne outbreaks due to these pathogens was estimated to be ~$7.7 billion in 2018 [6]. Aside from bacterial contamination, spoilage microorganisms, such as fungi, are also major food safety concerns in fresh produce. A common food spoilage fungus, *Aspergillus niger* (*A. niger*), causes storage mold and fruit rot and is commonly spread through fruit ripening and long-term storage, particularly at temperatures between 25 °C and 30 °C [7,8]. For these reasons, we carefully selected strains representing these microbial species for evaluation in this study. On the other hand, norovirus is the leading cause of foodborne illnesses and is responsible for 19–21 million cases of foodborne infections and 570–800 deaths annually in the US [9,10,11]. Due to the difficulty of establishing a cell culture system for the growth of human norovirus, and the testing of GS-2-induced neutralization, we instead utilized murine norovirus (MNV) as a surrogate virus for evaluation of the antiviral properties of GS-2 [12].

The contamination of fresh produce by pathogenic and spoilage microorganisms may occur at various pre- and post-harvest stages. Agricultural soil and irrigation water are among one of the top sources of contamination pre-harvest as both can serve as reservoirs for bacteria, fungi, and viruses [13,14]. Harvesting and steps during post-harvest such as manual sorting, packing, processing, shipping, and food preparation can also introduce bacteria, fungus, and viruses to fresh produce [15]. With new regulations mandating for reusable packaging, if the packaging is contaminated, it can cross contaminate new batches of produce [16]. Current industrial practice to decontaminate reusable packaging is washing and sanitizing with bleach, quaternary ammonium compounds, and/or peracetic acid [17,18]. However, these chemicals bring concerns of potential hazardous chemical residues, and such decontamination processes may not always be reliable due to the improper usage of sanitizing chemicals. On the other hand, biofilm formations can assist bacteria on produce surfaces in escaping treatments of disinfectants and sanitizers [18,19]. Therefore, developing new technologies to minimize pathogen transmission through reusable packaging is of the foremost importance. Wintermute Biomedical developed a novel chemistry, GS-2, to address this challenge by providing a water-soluble, food-safe antimicrobial coating composed of capric acid (fatty acid), L-arginine (amino acid), and thymol (monoterpene phenols) [20]. Capric acid, L-arginine, and thymol are considered safe by U.S. regulatory agencies such as the Food & Drug Administration for human consumption [21,22,23,24]. This makes GS-2 an ideal candidate for addressing the food safety challenges associated with reusable packaging.

The antimicrobial properties of fatty acids, including capric acid, have been well-documented, with research demonstrating their effectiveness against a broad spectrum of bacteria [25]. However, the inherent water-insolubility of fatty acids has historically limited their practical applications [26,27]. Wintermute Biomedical overcame this limitation by developing a patented process that converts fatty acids into water-soluble salts, enabling their use at various concentrations without organic solvents or solubilizers [20]. This improvement not only enhances the stability and efficacy of the antimicrobial agent, but also makes it compatible with existing spraying and application systems, allowing for easy adoption in the specialty crop industry [20]. Preliminary feasibility studies have demonstrated that GS-2 forms a clear, durable, and adherent film on glass surfaces, providing extended antimicrobial activity for at least 60 days after application [20]. The cost-effectiveness of GS-2, combined with its broad-spectrum antimicrobial activity against bacteria, fungi, and viruses, positions this chemical formulation as a promising solution for ensuring the microbiological safety of reusable packaging.

The overall goal of this study was to evaluate the efficacy of GS-2 for its practical use on select cardboard and plastic packaging materials in improving the microbiological safety of high-value U.S. specialty crops. Specifically, we evaluated the following: (1) the efficacy of GS-2 against *A. niger*, *E. coli* O157:H7, *L. monocytogenes*, *S. enterica*, and murine norovirus as a human norovirus surrogate at different exposure times; (2) different GS-2 concentrations against *A. niger*, *E. coli* O157:H7, *L. monocytogenes*, and *S*. *enterica*; (3) the efficacy of GS-2 under various produce storage conditions against *A. niger*, *E. coli* O157:H7, and *L. monocytogenes*; and (4) the efficacy of GS-2 against *A. niger*, *E. coli* O157:H7, *L. monocytogenes*, and *S. enterica* transfers from cardboard and plastic surfaces to grape tomatoes as a representative specialty crop.

## 2. Materials and Methods

### 2.1. Preparation of Microbial Inocula

Three bacterial strains (*E. coli* O157:H7 TW14359, *L. monocytogenes* LS810, *S. enterica* Agona 447967), one fungal strain (*A. niger* van Tieghem 16888), and one MNV strain (CW1) were used in this study. The three bacterial strains were implicated in previous human foodborne disease outbreaks: *E. coli* O157:H7 TW14359 was from the 2006 spinach outbreak, *L. monocytogenes* LS810 was from the 2011 cantaloupe outbreak, and *S. enterica* Agona 447067 was from the 1998 toasted oats cereal outbreak, respectively [28,29,30]. For long term storage, the bacterial strains were maintained at −80 °C in Tryptic Soy Broth (TSB, Difco, Becton Dickinson, Franklin Lakes, NJ, USA) supplemented with 20% glycerol (VWR International, Radnor, PA, USA). All three bacterial strains were rifampicin-resistant and maintained on Brain Heart Infusion Agar (BHIA, Difco, Becton Dickinson, NJ, USA) supplemented with 200 µg/mL Rifampicin powder (Chem-Inpex International, Dale, IL, USA) (BHIA^rif^) as working stocks to eliminate background microorganisms. *A. niger* was not rifampicin-resistant and was maintained on potato dextrose agar (PDA, Difco, Becton Dickinson, Franklin Lakes, NJ, USA) as a working stock. Working stocks were stored at 4 °C for a maximum of 4 weeks. For inoculation, the bacterial strains were prepared with overnight incubation at 37 °C in 10 mL TSB for 18 h. The overnight cultures were retrieved, 1 mL was taken and spun at 13,000× *g* for 1 min, and the supernatant was decanted. The resulting pellets were washed twice with 1 mL Butterfield’s Phosphate Buffer (BPB). BPB stock was prepared by dissolving 68 g of potassium phosphate (Difco, Becton Dickinson, Franklin Lakes, NJ, USA) in 1 L of sterile distilled water (sdH_2_O) and adjusted to pH 7.2. Subsequently, 1.25 mL of the dissolved potassium phosphate solution was added to 1 L of sterile distilled water to create BPB for use in this study. For *A. niger*, cultures were prepared 5 days prior to inoculation at 37 °C in 10 mL potato dextrose broth (PDB, Difco, Becton Dickinson, Franklin Lakes, NJ, USA). Working stocks of MNV were generated as previously reported [31] and viral inoculum was diluted in phosphate-buffered saline (PBS) to a concentration of 10^6^ PFU/50 µL.

### 2.2. Evaluation of Efficacy of GS-2 Against A. niger, E. coli O157:H7, L. monocytogenes, and S. enterica Agona on Plastic and Cardboard Coupons at Different Exposure Times

Plastic materials of acrylonitrile butadiene styrene (ABS), high-density polyethylene (HDPE), polypropylene (PP), and cardboard boxes from a local company were used in this study to represent common food packaging materials for the storage and transport of fresh produce. ABS, HDPE, PP, and cardboard were cut into 1 × 1-inch coupons, sanitized with 70% ethanol, and separated into control and treatment groups. For the treatment group, stock solutions of GS-2 (Wintermute Biomedical, Ten Carbon Chemistry, Scoresby, Victoria, Australia) were diluted with sdH_2_O to a concentration of 3%. After dilution, 0.1 mL of 3% GS-2 was pipetted onto the coupons of each plastic and cardboard coupon individually, spread with a plastic spreader, and then dried in a biosafety cabinet for 60 min. The plastic and cardboard coupons in both the control and treatment groups were then spot inoculated individually with 0.1 mL of *A. niger*, *E. coli* O157:H7, *L. monocytogenes*, or *S. enterica* Agona and then spread with a plastic spreader. The resulting inoculations were ~10^7^ CFU/mL for *A. niger* and ~10^9^ CFU/mL for the bacteria. After 15 min and 60 min, the plastic and cardboard coupons were placed in a sterile Whirlpak bag filled with 5 mL BPB and stomached for 1 min at 150 rpm for viable cell recovery. Serial dilutions in BPB were performed and plated on BHIA^rif^ for bacterial strains and PDA was for *A. niger*, respectively. *E. coli* O157:H7 and *S. enterica* Agona plates were incubated at 37 °C for 24 h and *A. niger* and *L. monocytogenes* plates were incubated for 48 h at 37 °C prior to viable cell enumeration.

### 2.3. Evaluation of Efficacy of GS-2 Against MNV on Plastic and Cardboard Coupons at Different Exposure Times

One hundred µL or 150 µL of 3% GS2 in H_2_O and a water control were added to the plastic (ABS and PP) or cardboard coupons, respectively. The coating was dried in a biosafety cabinet for 60 min, before the addition of 1 × 10^6^ PFU MNV in 50 µL PBS. The squares were then placed in a closed box containing wet paper towels, to decrease virus loss by evaporation. For the ABS and PP coupons, at the treatment end point, 950 µL of serum-free DMEM (Gibco, Grand Island, NY, USA) was used to recover MNV. Samples were centrifuged at 10,000× *g* for 10 min at 4 °C, then supernatant was transferred to a new microcentrifuge tube and frozen prior to titration via plaque-forming assay. For the cardboard coupons, at the treatment end point, the coupons were cut into small fragments and placed in 10 mL serum-free DMEM. The samples were frozen, thawed, vortexed for 10 s, and rolled for 10 min. Samples were centrifuged at 4000× *g* for 10 min at 4 °C, then supernatant was transferred to a new microcentrifuge tube prior to titration via plaque-forming assay.

### 2.4. Evaluation of Different GS-2 Concentrations Against A. niger, E. coli O157:H7, L. monocytogenes, and S. enterica Agona on Plastic and Cardboard Coupons

Plastic and cardboard coupons were prepared as discussed in 2.2. GS-2 was diluted with sdH_2_O to concentrations of 0.3%, 1%, and 3% for bacteria and 3%, 3.5%, and 4% for *A. niger*. For all concentrations of GS-2, 0.1 mL was pipetted onto the coupon of the plastic and cardboard coupons separately, spread with a plastic spreader and then dried in a biosafety cabinet for 60 min. The plastic and cardboard coupons in both the control and treatment groups were then spot inoculated individually with 0.1 mL of *A. niger*, *E. coli* O157:H7, *L. monocytogenes*, or *S. enterica* Agona and then spread with a plastic spreader. After 60 min, the plastic and cardboard coupons were placed in a sterile Whirlpak bag filled with 5 mL BPB and stomached for 1 min at 150 rpm for viable cell recovery. Serial dilutions in BPB were performed and then plated on BHIA^rif^ for bacterial strains and PDA for *A. niger*. *E. coli* O157:H7 and *S. enterica* Agona plates were incubated at 37 °C for 24 h and *A. niger* and *L. monocytogenes* plates were incubated for 48 h prior to enumeration.

### 2.5. Evaluation of A. niger, E. coli O157:H7, L. monocytogenes, and S. enterica Agona Transfer from GS-2 Treated Plastic and Cardboard to Grape Tomatoes

Plastic and cardboard coupons, along with GS-2, were prepared as discussed in 2.2. After treatment with GS-2, all coupons were dried for 60 min in a biosafety cabinet. The other half of plastic and cardboard coupons were untreated to serve as controls. Treated and untreated coupons were inoculated with 0.1 mL of *A. niger*, *E. coli* O157:H7, *L. monocytogenes*, or *S. enterica* Agona cultures and were dried for another 60 min in a biosafety cabinet. Grape tomatoes purchased from a local grocery store were placed on top of the plastic and cardboard coupons for 30 min. After 30 min, the plastic and cardboard coupons and grape tomatoes were individually placed in a sterile Whirlpak bag filled with 5 mL BPB and stomached for 1 min at 150 rpm for viable cell recovery. Serial dilutions in BPB were performed and then plated on BHIA^rif^ for bacterial strains and PDA for *A. niger*. *E. coli* O157:H7 and *S. enterica* Agona plates were incubated at 37 °C for 24 h and *A. niger* and *L. monocytogenes* plates were incubated for 48 h prior to population enumeration.

### 2.6. Efficacy of GS-2 Against A. niger, E. coli O157:H7, and L. monocytogenes on Plastic and Cardboard Coupons After Storage

Plastic and cardboard coupons, along with GS-2, were prepared as discussed in 2.2. Treatment group plastic and cardboard coupons were treated with 3% GS-2. For *A. niger*, both the plastic and cardboard coupons were treated with 3% GS-2. After treatment with GS-2, all coupons were dried for 60 min in a biosafety cabinet. Treated plastic and cardboard coupons were stored in two representative storage conditions for up to 42 days, at (1) 4 °C at 90% relative humidity for cold storage and (2) 18 °C at 45% relative humidity for ambient storage. At day 0, 2, 7, 14, and 42, the treated plastic and cardboard coupons were removed from storage and inoculated with 0.1 mL of *A. niger*, *E. coli* O157:H7, and *L. monocytogenes* cultures. After 60 min, the plastic and cardboard coupons were placed in a sterile Whirlpak bag filled with 5 mL BPB and stomached for 1 min at 150 rpm for viable cell recovery. Serial dilutions in BPB were performed and then plated on BHIA^rif^ for bacteria strains and PDA for *A. niger*. *E. coli* O157:H7 plates were incubated at 37 °C for 24 h and *A. niger* and *L. monocytogenes* plates were incubated for 48 h prior to enumeration.

### 2.7. Quantification of MNV Titre by Plaque-Forming Assay

For the titration of MNV after recovery from plastic and cardboard squares, RAW264.7 cells were cultured in 12-well tissue culture plates overnight to reach 100% confluency. Recovered virus was then serially diluted in serum-free Dulbecco’s Modified Eagle Medium (DMEM, Gibco, NY, USA) and applied to the cells. Following a 1 h incubation at 37 °C, virus inoculum was removed and a 2 mL overlay (70% DMEM, 2.5% [vol/vol] Foetal Calf Serum (FCS, Gibco, NY, USA), 13.3 mM NaHCO_3_ (Gibco, Grand Island, NY, USA), 22.4 mM HEPES (Gibco, NY, USA), 200 mM GlutaMAX (Gibco, Grand Island, NY, USA), and 0.35% [wt/vol] SeaPlaque agarose (Lonza, Cohasset, MN, USA)) was added. Cells were placed at 4 °C for 30 min to solidify the agarose overlay, then incubated at 37 °C for 48 h. Monolayers were fixed with 10% neutral buffered formalin (Trajan, Victoria, Australia) for 30 min at room temperature and then the overlay was removed. Fixed cell monolayers were stained with 1 mL Toluidine blue (ChemSupply Australia, Gillman SA, Australia) for 30 min, then washed with water. Plaque-forming units per mL (PFU/mL), or infectivity, were determined by the enumeration of plaques.

### 2.8. Statistical Analysis

Each treatment was conducted with three biological replicates and three technical replicates for each strain tested. A total of 4284 data points were collected for statistical analysis. Results of *A. niger*, *E. coli* O157:H7, *L. monocytogenes*, and *S. enterica* Agona populations from 2.2 were converted to log values and analyzed using paired *t*-tests to determine significant differences (*p* < 0.05) compared to each control using GraphPad Prism, version 10.3.1 (GraphPad Software, LLC, Boston, MA, USA). For 2.3, MNV plaque-forming units were converted to log values and analyzed using paired *t*-tests to determine significant differences (*p* < 0.05) compared to the control. For 2.4–2.6, *A. niger*, *E. coli* O157:H7, *L. monocytogenes*, and *S. enterica* Agona populations were converted to log values and analyzed using a one-way analysis of variance (one-way ANOVA), and Turkey’s honestly significant difference (Turkey’s HSD) was conducted for means separation with RStudio, version 1.1463 (RStudio, Inc., Boston, MA, USA). *p*-values of <0.05 were considered statistically significant.

## 3. Results

### 3.1. Efficacy of GS-2 Against Bacterial, Fungal, and Viral Populations After 15 and 60 min Exposure

**Figure 1:**
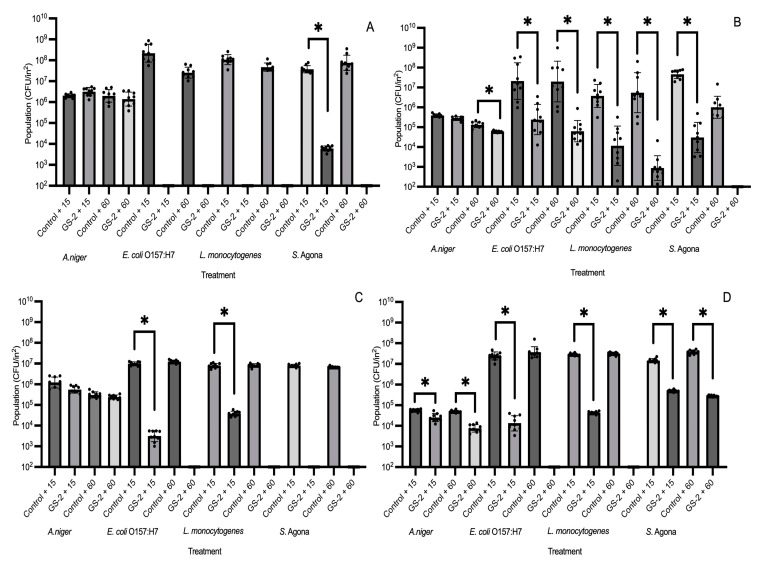
Population changes (CFU/in^2^) of *A. niger*, *E. coli* O157:H7, *L. monocytogenes*, and *S. enterica* Agona (*S.* Agona) on 3% GS-2 treated or non-treated (control) acrylonitrile butadiene styrene (**A**), cardboard (**B**), high-density polyethylene (**C**), and polypropylene (**D**) coupons after 15 or 60 min of exposure time. Limit of detection is 2 log CFU/in^2^. Values are presented as mean ± standard deviation. Asterisks (*) represent significant differences between treatments (control + 15 vs. GS-2 + 15 or control + 60 vs. GS-2 + 60) within *A. niger* or bacterial strains.

Cardboard and three different plastic coupons (ABS, HDPE and PP) were first surface treated with 3% GS-2 and then inoculated with *A. niger*, *E. coli* O157:H7, *L. monocytogenes*, or *S. enterica* Agona separately. After an exposure time of 15 or 60 min, microbial populations were enumerated. On plastic coupons (ABS, HDPE, and PP), 60 min exposure on 3% GS-2-treated coupons reduced populations of *E. coli* O157:H7 and *L. monocytogenes* to below the detection limit, suggesting a reduction of >5 log CFU/in^2^ compared to the control (Figure 1A,C,D). For *S. enterica* Agona, 60 min of exposure time reduced populations to below the detection limit, with a reduction of >5 log CFU/in^2^ on ABS, cardboard, and HDPE coupons compared to the control (Figure 1A–C). On PP coupons, both 15 and 60 min exposure were unable to reduce *S. enterica* Agona populations to below the detection limit, but both were able to significantly reduce *S. enterica* Agona populations by 1.7–2 log CFU/in^2^ compared to the control (*p* < 0.06, Figure 1D). An exposure of 15 min was able to reduce *E. coli* O157:H7 and L. monocytogenes populations to below the detection limit with a >5 log CFU/in^2^ reduction on ABS coupons, but 15 min was less effective for both strains on HDPE and PP coupons and reduced populations by ~2.5–4 log CFU/in^2^ compared to the control (*p* < 0.05, Figure 1A,C,D). On cardboard coupons, both 15 and 60 min were less effective against *E. coli* O157:H7 and *L. monocytogenes* compared to the plastic coupons, resulting in reductions of 1–2.9 log CFU/in^2^ (*p* < 0.05, Figure 1B). In comparison, both exposure times of 15 and 60 min were not effective against *A. niger* on all tested coupons as 3% GS-2 was unable to significantly reduce the *A. niger* population on both ABS and HDPE (*p* > 0.05) and an exposure time of 60 min only reduced the *A. niger* population by ~0.3 log CFU/in^2^ and ~1 log CFU/in^2^ on cardboard and PP coupons, respectively (*p* < 0.05, Figure 1A–D). The effectiveness of 3% GS-2 against *A. niger* and the three bacteria strains were dependent on the coupon, but the results supported that an exposure time of 60 min was more effective at reducing bacterial populations.

**Figure 2:**
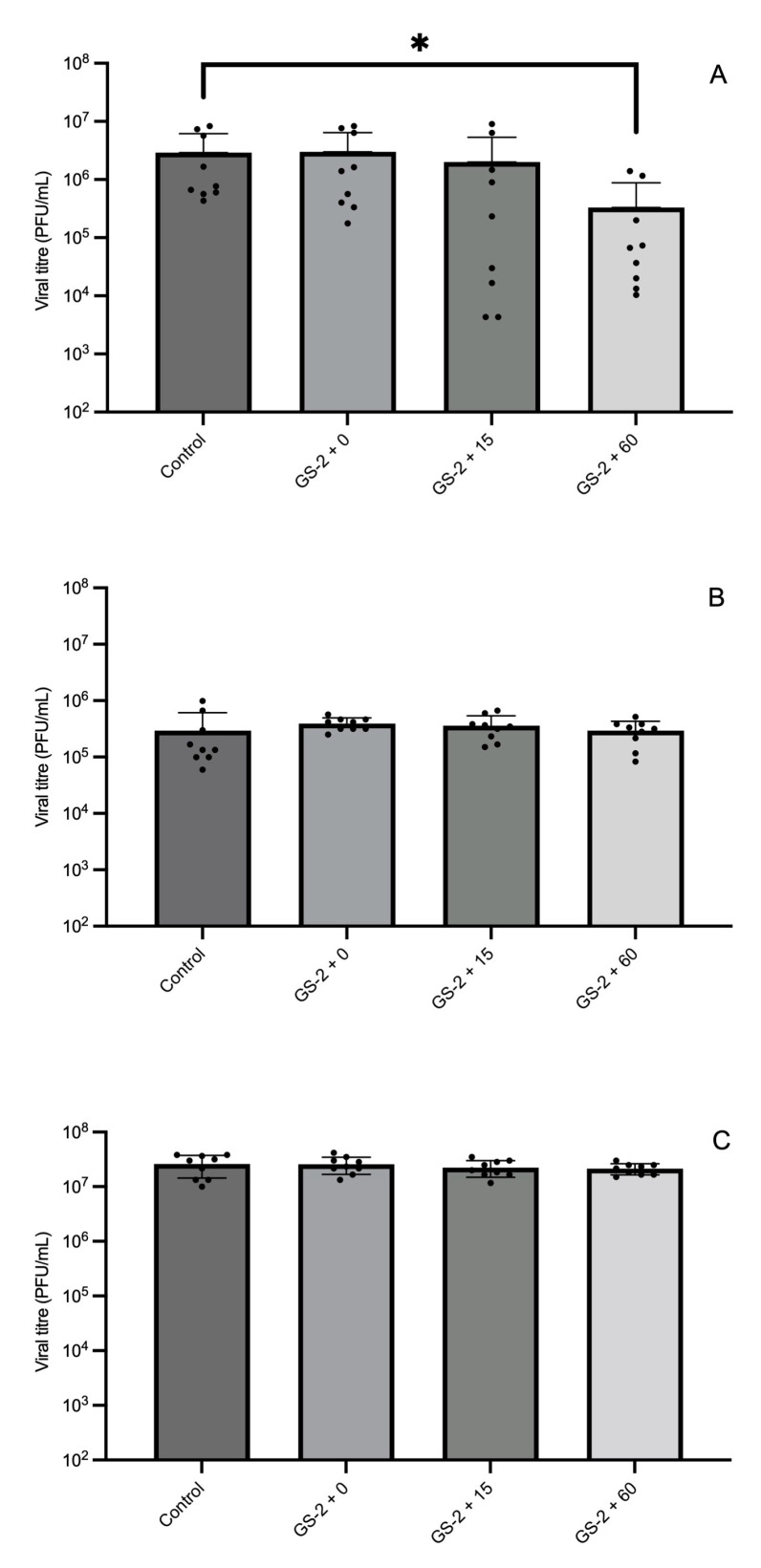
Recovery of murine norovirus (PFU/mL) with no-GS-2 treatment (control) and 3% GS-2 treatment for 0 (GS-2 + 0), 15 (GS-2 + 15), or 60 (GS-2 + 60) minutes on acrylonitrile butadiene styrene (**A**), cardboard (**B**), and polypropylene (**C**) coupons. Limit of detection is 2 log PFU/mL. Values are presented as mean ± standard deviation. Asterisks (*) represent significant differences between treatments (GS-2 + 0, GS-2 + 15, or GS-2 + 60 vs. control) on coupon.

To determine the efficacy of GS-2 against MNV and the most optimal GS-2 exposure time, cardboard and two different plastic coupons (ABS and PP) were first treated with 3% GS-2, before drying in a biosafety cabinet for 60 min, then inoculated with MNV. After an exposure time of 0 min, 15 min, or 60 min, viral titers were quantified via plaque-forming assay. GS-2 was more effective against MNV on ABS coupons compared to cardboard and PP coupons. On ABS coupons, when MNV was exposed to GS-2 for 60 min, MNV titers were significantly reduced by 1 log PFU/mL compared to the control (*p* < 0.05, Figure 2A). In contrast, an exposure time of 15 min and 0 min were not significantly different compared to the control (*p* > 0.05, Figure 2A). On cardboard and PP coupons, regardless of the exposure time (0, 15, or 60 min), there was no significant reduction in MNV titers after GS-2 treatment (*p* > 0.05, Figure 2B,C).

### 3.2. Evaluation of Different GS-2 Concentrations for Efficacy in Reducing Bacterial Populations

**Figure 3:**
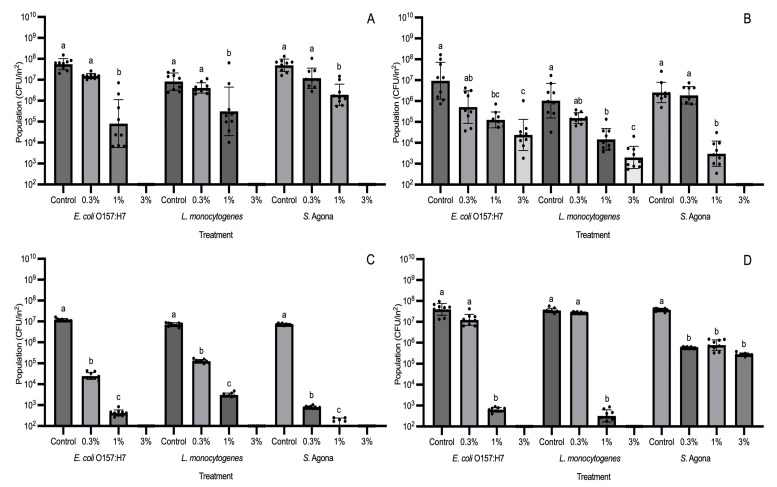
Population changes (CFU/in^2^) of *E. coli* O157:H7, *L. monocytogenes*, and *S. enterica* Agona on GS-2-treated coupons at concentrations 0.3%, 1%, and 3% or with no GS-2 (control). Four coupons included acrylonitrile butadiene styrene (**A**), cardboard (**B**), high-density polyethylene (**C**), and polypropylene (**D**). Limit of detection is 2 log CFU/in^2^. Values are presented as mean ± standard deviation. Different superscripts (a–c) represent significant differences between treatments within bacterial strain.

GS-2 was prepared in three different concentrations (0.3%, 1%, and 3%) and inoculated on ABS, cardboard, HDPE, and PP coupons. The GS-2-treated coupons were then inoculated with *E. coli* O157:H7, *L. monocytogenes*, or *S. enterica* Agona separately. After an exposure time of 60 min, populations were enumerated. On all three plastic coupons, 3% GS-2 reduced *E. coli* O157:H7 and *L. monocytogenes* populations to below the detection limit, resulting in a population reduction of >5 log CFU/in^2^ (Figure 3A,C,D). For 1% GS-2 on the three plastic coupons, populations of *E. coli* O157:H7 and *L. monocytogenes* were significantly reduced by ~2.5–4.4 log CFU/in^2^ (*p* < 0.05, Figure 3A,C,D). When 0.3% GS-2 was applied to the three plastic coupons, *E. coli* O157:H7 and *L. monocytogenes* populations were not significantly different compared to the control on ABS and PP (*p* > 0.05, Figure 3A,D); however, *E. coli* O157:H7 and *L. monocytogenes* populations were significantly reduced by ~2–2.5 log CFU/in^2^ on HDPE coupons (*p* < 0.05, Figure 3C). It was only on cardboard coupons where 3% GS-2 was unable to reduce *E. coli* O157:H7 and *L. monocytogenes* populations to below the detection limit and instead significantly reduced populations by ~2.5 log CFU/in^2^ (*p* < 0.05, Figure 3B). In contrast, 3% GS-2 was able to reduce *S. enterica* Agona populations to below the detection limit on cardboard coupons, resulting in a population reduction of >4.7 log CFU/on^2^ (Figure 3B). This trend continued on both ABS and HDPE coupons where 3% GS-2 reduced *S. enterica* Agona populations to below the detection limit for a reduction of >5 log CFU/in^2^ (Figure 3A,C). However, 3% GS-2 was least effective against *S. enterica* Agona populations on PP coupons, but with a significant reduction of ~2 log CFU/in^2^ (*p* < 0.05). A similar trend was observed for 0.3% and 1% GS-2 as *S. enterica* Agona populations were not significantly different between 0.3%, 1%, and 3% GS-2 treatments on PP coupons (*p* > 0.05, Figure 3D). The results show that 3% GS-2 is more effective than lower concentrations (0.3% and 1%); however, the effectiveness is dependent on the bacterial strain and the coupon. For the lower concentrations, 1% GS-2 may be less effective at reducing bacterial populations compared to 3% GS-2, but it was still able to significantly reduce populations by ~1.5–4.4 log CFU/in^2^ (*p* < 0.05), while 0.3% GS-2 did not significantly reduce *E. coli* O157:H7 and *L. monocytogenes* populations on ABS, cardboard, and PP coupons (*p* > 0.05). Therefore, if GS-2 is used in the industry, we recommend that at least 1% GS-2 should be used, whereas 3% GS-2 would be the more effective against bacteria.

#### Evaluation of Effective GS-2 Concentrations for Reducing Fungal Populations

**Figure 4:**
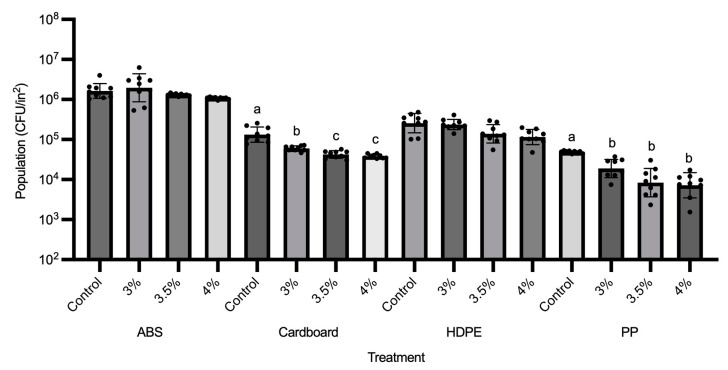
Population changes (CFU/in^2^) of *Aspergillus niger* on GS-2-treated coupons at concentrations of 3%, 3.5%, and 4% compared to no GS-2 (control). Coupons tested included acrylonitrile butadiene styrene, cardboard, high-density polyethylene, and polypropylene. Limit of detection is 2 log CFU/in^2^. Values are presented as mean ± standard deviation. Different superscripts (a–c) represent significant differences between treatments on same coupon.

From the results of Figure 1, 3% GS-2 at the longer exposure time of 60 min was not as effective at reducing *A. niger* populations compared to bacterial populations. For this reason, higher concentrations at 3.5% and 4% were tested for *A. niger*. The only coupons where *A. niger* populations were significantly reduced after GS-2 treatment were cardboard and PP (Figure 4). The result was similar to the trend showcased in Figure 1, in which *A. niger* populations were reduced after GS-2 treatment on cardboard and PP coupons. On cardboard, 3% GS-2 reduced *A. niger* populations by ~0.3 log CFU/in^2^ (*p* < 0.05), whereas *A. niger* populations after 3.5% and 4% GS-2 treatment were not significantly different from each other (*p* > 0.05). Treatment with 3.5 and 4% GS-2 further reduced *A. niger* populations by ~0.1 log CFU/in^2^ compared to 3% GS-2, and by ~0.4 log CFU/in^2^ compared to the control (*p* < 0.05) (Figure 4). On PP coupons, *A. niger* populations after 3%, 3.5%, and 4% GS-2 treatment were not significantly different from each other (*p* > 0.05), but all were significantly reduced compared to the control, by ~0.6 log CFU/in^2^ (*p* < 0.05, Figure 4). The results suggested that the effectiveness of GS-2 against *A. niger* was on the opposite spectrum of its effectiveness against bacteria. On the coupons where there was significant *A. niger* population reduction after GS-2 treatment (*p* < 0.05), the population reduction is under 1 log, between ~0.3 and 0.6 log CFU/in^2^ (Figure 1 and Figure 4).

### 3.3. Efficacy of GS-2 Against Bacterial and Fungal Population Transfers to Grape Tomatoes

**Figure 5:**
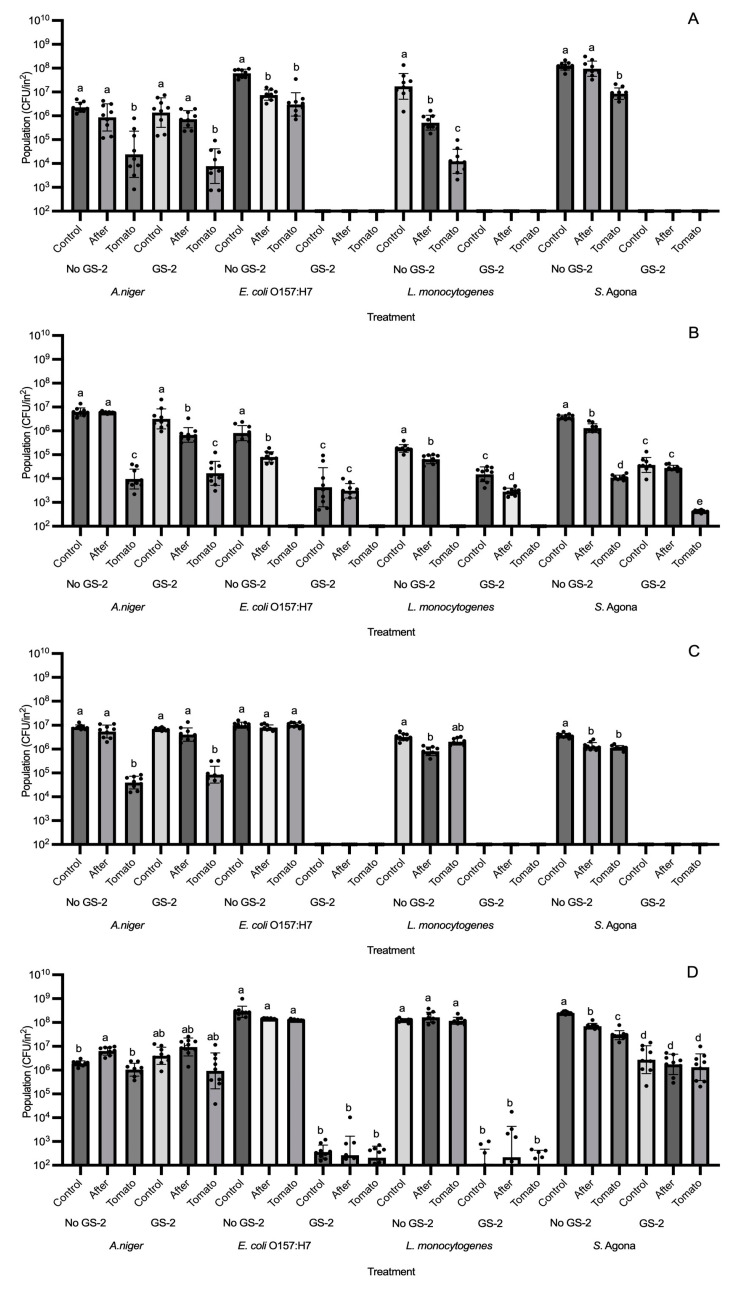
Population (CFU/in^2^) of *A. niger*, *E. coli* O157:H7, *L. monocytogenes*, and *S. enterica* Agona transferred from no-GS-2-treated and 3% GS-2-treated acrylonitrile butadiene styrene (**A**), cardboard (**B**), high-density polyethylene (**C**), and polypropylene (**D**) coupons to grape tomatoes. Treatment groups include *A. niger* or bacterial populations left on coupons immediately after *A. niger* or bacterial inoculation (control), *A. niger* or bacterial populations left on coupons after transfer to grape tomatoes (After), and *A. niger* or bacterial populations transferred onto grape tomato (Tomato). Limit of detection is 2 log CFU/in^2^. Values are presented as mean ± standard deviation. Different superscripts (a–d) represent significant differences between treatments within *A. niger* or bacterial strain.

Coupons were first inoculated with 3% GS-2 and then inoculated with *A. niger*, *E. coli* O157:H7, *L. monocytogenes*, or *S. enterica* Agona separately. A grape tomato was placed on top of each coupon and afterwards, *A. niger* and bacterial populations were enumerated on both coupons and tomato surfaces. On both ABS and HDPE coupons, GS-2 reduced *E. coli* O157:H7, *L. monocytogenes*, and *S. enterica* Agona populations to below the detection limit. For this reason, bacterial populations on the grape tomato and on the coupons were both below the detection limit (Figure 5A,C). On PP coupons, there were no significant differences between *E. coli* O157:H7, *L. monocytogenes*, and *S. enterica* Agona populations inoculated on the coupon (control), after transfer to grape tomato (After), and transferred onto grape tomato (Tomato) (*p* > 0.05, Figure 5D), but were significantly decreased compared to no GS-2 by ~5–5.8 log CFU/in^2^ for *E. coli* O157:H7 and *L. monocytogenes* and ~1.7–2 log CFU/in^2^ for *S. enterica* Agona (*p* < 0.05, Figure 5D). On cardboard coupons, there was a lower reduction in bacterial populations after GS-2 treatment compared to ABS, HDPE, and PP coupons as *E. coli* O157:H7, *L. monocytogenes*, and *S. enterica* Agona populations were significantly reduced by ~1.2–2.2 log CFU/in^2^, ~1 log CFU/in^2^, and ~1.5–2 log CFU/in^2^, respectively (*p* < 0.05, Figure 5B). For *A. niger*, there is limited effectiveness from GS-2 treatment compared to bacteria. After GS-2 treatment, there were no significant differences between the no-GS-2 and GS-2 treatments (*p* > 0.05, Figure 3A–D).

### 3.4. Efficacy of GS-2 During 42 Days of Storage at 4 °C and 90% RH and 18 °C and 45% RH Against Bacterial and Fungal Populations

**Figure 6:**
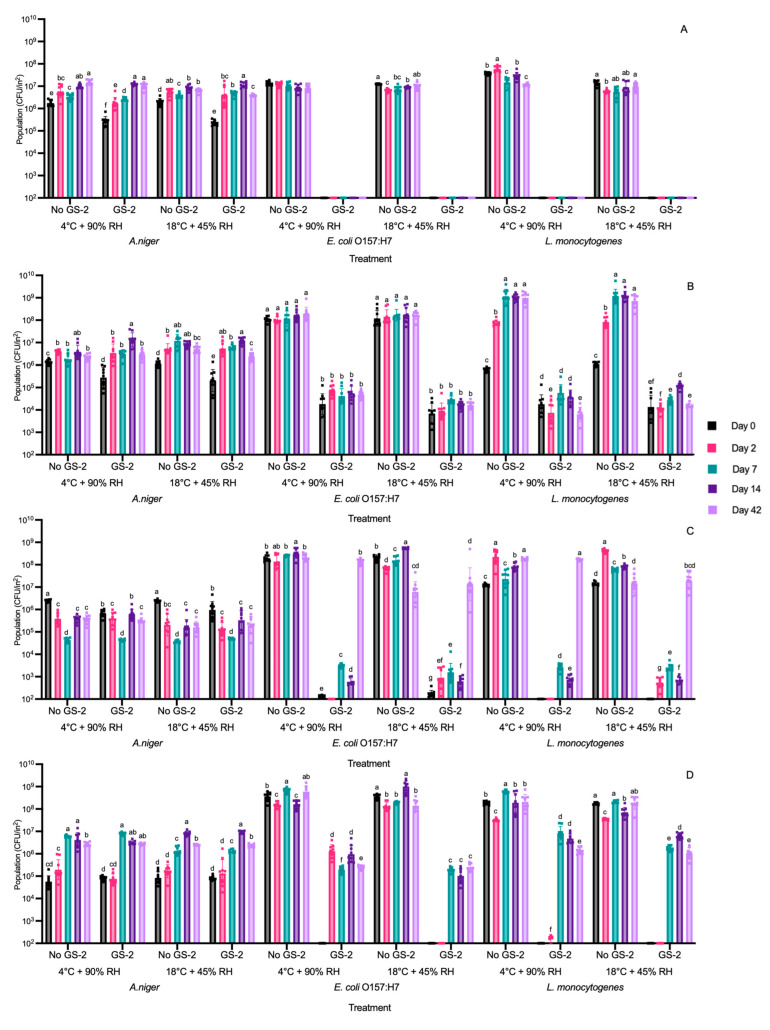
Populations (CFU/in^2^) of *A. niger*, *E. coli* O157:H7 and *L. monocytogenes* on 3% GS-2- and non-GS-2 (no-GS-2)-treated acrylonitrile butadiene styrene (**A**), cardboard (**B**), high-density polyethylene (**C**), and polypropylene (**D**) coupons stored at 4 °C and 90% relative humidity (RH) and 18 °C and 45% RH for 0, 2, 7, 14, and 42 days, respectively. Limit of detection is 2 log CFU/in^2^. Values are presented as mean ± standard deviation. Different superscripts (a–g) represent significant differences between no GS-2 and GS-2 for same storage conditions (4 °C and 90% RH or 18 °C and 45% RH) for *A. niger* or bacterial strains.

Coupons were first inoculated with 3% GS-2 and then inoculated with *A. niger*, *E. coli* O157:H7, or *L. monocytogenes* separately. The coupons were stored for up to 42 days under cold (4 °C and 90% RH) or ambient (18 °C and 45% RH) storage conditions. On days 0, 2, 7, 14, and 42, the coupons were retrieved, and microbial populations were enumerated. On both ABS and cardboard coupons, the bactericidal effectiveness of GS-2 throughout the 42 d storage period for both storage conditions remained unchanged (Figure 6A,B).

Specifically, on ABS coupons, GS-2 reduced *E. coli* O157:H7 and *L. monocytogenes* populations to below the detection limit, reducing populations by >5 log CFU/in^2^ on day 0 and across the storage days up to day 42 (Figure 6A). By day 42, GS-2 was still effective by keeping *E. coli* O157:H7 and *L. monocytogenes* populations below the detection limit (Figure 6A). On cardboard coupons, the populations of *E. coli* O157:H7 and *L. monocytogenes* were decreased by ~2–4 log CFU/in^2^ on day 0 for both storage conditions and GS-2 remained effective at day 42 as *E. coli* O157:H7 and *L. monocytogenes* populations were not significantly different (*p* > 0.05) or significantly decreased by ~0.5 log CFU/in^2^ compared to the populations at day 0 (*p* < 0.05) (Figure 6B).

On HDPE and PP coupons, a decrease in GS-2 effectivity was shown at longer storage periods. On HDPE, *E. coli* O157:H7 and *L. monocytogenes* populations were significantly reduced by ~4–6 log CFU/in^2^ (*p* < 0.05) or reduced to below the detection limit on days 0–14 for both storage conditions (Figure 6C). However, at day 42, *E. coli* O157:H7 and *L. monocytogenes* populations were not significantly different compared to the control (*p* > 0.05, Figure 6C), indicating that the efficacy of GS-2 had lessened significantly at some time between day 14 and 42 and may no longer be effective. In contrast, when PP coupons with GS-2 were stored at both storage conditions, *E. coli* O157:H7 and *L. monocytogenes* populations were reduced to below the detection limit on day 0, with a reduction of >6 log CFU/in^2^, and populations remained below the detection limit on day 2 for the PP coupons stored at 18 °C and 45% RH. For PP coupons stored at 4 °C and 90% RH, *E. coli* O157:H7 and *L. monocytogenes* populations were significantly reduced by ~2 log CFU/in^2^ and ~5.4 log CFU/in^2^, respectively (*p* < 0.05, Figure 6D). From day 7–42, *E. coli* O157:H7 and *L. monocytogenes* populations were significantly reduced by GS-2 for both storage conditions to be within the range of ~2.5–4 log CFU/in^2^ (*p* < 0.05, Figure 6D). This indicates that while GS-2 efficacy decreased when compared to day 0, GS-2 was stable for both storage conditions from day 7 to day 42 (Figure 6D). The efficacy of GS-2 against *A. niger* during both storage conditions shows a similar trend compared to the earlier results. When comparing both storage conditions to the same condition with no GS-2, populations were not significantly different (*p* > 0.05), with reductions < 0.7 log CFU/in^2^ for all coupons (*p* < 0.05, Figure 6A–D). The results collectively showed that GS-2 retained its effectiveness against *E. coli* O157:H7 and *L. monocytogenes* up to 14–42 days, and the efficacy varied among the different packaging materials used.

## 4. Discussion

Overall, our study demonstrated that GS-2, a food-safe antimicrobial formulation which comprises capric acid (10-carbon fatty acid), L-arginine (amino acid), and thymol (monoterpene phenol) [20], was highly effective to inactivate bacterial pathogens on various reusable packaging materials; however, the antimicrobial efficacy of GS-2 against spoilage fungus *A. niger* was rather limited. It is worth noting that some earlier studies suggested the potential use of other fatty acids, amino acids, and monoterpene phenols as antifungal agents based on challenge studies using liquid cultures [32]. For instance, three hydroxy unsaturated fatty acids (coriolic acid, linoleic acid, and oleic acid) were shown to be able to inhibit *A. niger* in liquid culture medium at minimum inhibitory concentrations (MICs) of 0.29 ± 0.07 g/L, 3.67 ± 1.53 g/L, and ≥8 g/L, respectively [33]. Coriolic acid and linoleic acid along with three other hydroxy fatty acids (13-hydroxy-12-octadecenoic acid, 10-hydroxy-12-octadecenoic acid, and ricinoleic acid) were found to be most effective with an MIC of 0.25 ± 0.00 g/L against *A. niger* in liquid culture, followed by coriolic acid with 0.26 ± 0.09 g/L, ricinoleic acid with 0.29 ± 0.10 g/L, 10-hydroxy-12-octadecenoic acid with 0.42 ± 0.13 g/L, and, lastly, linoleic acid with 4.00 ± 0.00 g/L [34]. Coriolic, ricinoleic, oleic, and linoleic acid were also used in a separate study against *A. niger* liquid culture but had higher MICs of 0.7 ± 0.2 g/L, 2.4 ± 0.0 g/L, >20 g/L, and 4.0 ± 0.0 g/L, respectively [35]. An accumulation of mono-, di-, and trihydroxy fatty acids from *Pseudomonas* 42A2 and linoleic acid had an MIC of 0.14 g/L against *A. niger* in liquid culture [36].

Amino acids have also been reported for antifungal properties in previous research [37]. One study suggested amino acid derivatives of L-Aspartic acid, D-Aspartic acid, L-Tyrosine, and D-Tyrosine were more effective against *A. niger* KCCM 11239, with MICs of 0.004 g/L, compared to L-Phenylalanine and L-Cysteine [38]. The third component of GS-2, thymol, has shown inhibition towards *A. niger* in a combination of essential oils which included thymol [39]. At a concentration of 5 µL/mL, the combination of essential oils including thymol was able to inhibit *A. niger* on Petri dishes through colony diameter measurements up to 9 days [39]. Another study found thymol at an MIC of 0.25 g/L to also inhibit *A. niger* when grown on Petri dishes [7]. Thymol, in comparison to other monoterpene phenols such as carvacrol, was found to be more effective at inhibiting *A. niger* culture in both pure liquid and in an encapsulated format over 30 days [40].

GS-2 has been previously tested against *Candida auris* (*C. auris*), a fungus in liquid culture, and the minimum fungicide concentration of 0.15 g/L was similar to the minimum bactericidal concentration of *Clostridium difficile* (*C. difficile*), a Gram-positive bacteria, suggesting that GS-2 is potent against fungal species when used in liquid cultures [20]. The cell walls of both *Candida* and *Aspergillus* species are made up of chitin, ß-1,6-glucan, ß-1,3-glucan, and GPI-anchored protein; however, *Aspergillus* species have higher cross-links between the glucans and the chitin fibers, which adds to the complexity of its structure [41,42]. *Aspergillus* species also have chitin in the outer layers of the cell wall, which increases its robustness and, in comparison, *Candida* species have a more dynamic and less rigid cell wall [41,42,43,44]. Scanning electron microscopy has shown that when thymol is implemented at the MIC against *A. niger*, cell deformation can be observed due to cell membrane destruction and the loss of cell wall strength [7]. Since the cell wall of *Candida* species is less rigid compared to *Aspergillus* species, it might be easier for GS-2, which contains thymol, to attack *C. auris*. We expected that a higher concentration of GS-2 would be required to achieve the same cell deformation in *A. niger* than in *C. auris*.

With fatty acids, the mode of action against bacteria and fungus generally occurs through destabilizing the cell membrane, increasing cell permeability to leak cytosolic content and with eventual cell lysis [25,45,46]. For amino acids such as L-arginine, the mode of action is similar to that of fatty acids and thymol, where it causes cell membrane permeability, and poly-l-arginine has been found to enhance membrane permeability more than poly-l-lysine [47,48,49,50]. Since the mode of actions of capric acid, L-arginine, and thymol all include cell membrane permeability and destruction, we assumed that this can be part of the mode of action of GS-2. It is worth noting that all previous research on the efficacy of fatty acids, amino acids, and monoterpene phenols against *A. niger* was carried out using either liquid culture or on the colonies directly on a Petri dish, the results of which cannot be directly translated to the realistic applications of such compounds for antimicrobial package coating. In our study, *A. niger* culture was directly inoculated on either a cardboard or plastic coupon before GS-2 treatment. It was not surprising that GS-2 was less effective at inactivating fungal cells on dry packaging materials than in pure liquid cultures. Moreover, under low-humidity conditions, *A. niger* can activate a stress response in the cell wall by upregulating the synthesis of chitin [51]. With an increase in chitin, it strengthens and increases the integrity of the cell wall, potentially reducing the efficacy of GS-2 in compromising the cell membrane.

Bacterial pathogens such as *E. coli* O157:H7, *L. monocytogenes*, and *S. enterica* do not have the mechanisms used by fungi to increase cell wall integrity under low moisture stress [52,53,54,55,56]. Instead, *E. coli* O157:H7 and *S. enterica* can respond to adverse environmental conditions through mechanisms such as biofilm production to create a microenvironment to preserve moisture, producing osmoprotectants such as trehalose to help stabilize proteins and cellular components [54,55,56,57]. The Gram-positive bacterium *L. monocytogenes*, in contrast, has a thicker peptidoglycan layer, which helps maintain cell integrity when the cell is exposed to stressful environmental conditions [58]. Aside from the thicker peptidoglycan layer, *L. monocytogenes* can also produce osmoprotectants to help retain moisture, similar to *E. coli* O157:H7 and *S. enterica*, and induce stress response proteins to survive desiccation [52,53,59]. Since bacteria do not modify their cell wall, this could potentially explain why GS-2 was more effective against bacteria than *A. niger* on different packaging surfaces in this study. However, further research to compare microbial stress response mechanisms between liquid culture and dry packaging materials under GS-2 treatment could help elucidate the varied GS-2 efficacy against different pathogenic and spoilage microorganisms.

The use of advanced imaging technologies such as scanning electron microscopy may help to further explore the correlation between the surface topography and characteristics of different packaging materials and the efficacy of GS-2 against microorganisms. For example, GS-2 was less effective for reducing *E. coli* O157:H7, *L. monocytogenes*, and *S. enterica* Agona populations on cardboard than on plastic coupons. One explanation could be that cardboard which is made out of cellulose fibers is relatively more porous and absorbent compared to plastics [60,61]. When GS-2 is added onto cardboard coupons, it can be absorbed into the material, potentially leaving less active chemicals and moisture on the surface to interact with the bacteria. Some bacterial cells could also be settled or trapped within the cellulose fibers, resulting in the lower bacterial transfer onto grape tomato that was shown in Figure 5B. For non-GS-2-treated cardboard, there was a small transfer of *E. coli* O157:H7 and *L. monocytogenes* onto grape tomatoes, whereas ~5.5 log CFU/in^2^ remained on the cardboard after transfer (Figure 5B). This was in contrast to the other plastic coupons, as all non-GS-2-treated plastic coupons transferred ~6–8 log CFU/in^2^ of *E. coli* O157:H7 and *L. monocytogenes* onto grape tomato (Figure 5A,C,D). These findings were similar to previous research where it was found that fruits packed in plastic showed a ~0.8 probability of higher contamination frequency compared to cardboard after 24 h and 48 h at 24 °C [60]. It was reported that when bacteria were entrapped in the cellulose fibers of cardboard, *E. coli* and *Saccharomyces cerevisiae* (*S. cerevisiae*) cells were subjected to more rapid lyses due to the absence of water and nutrients, but rapid lysis was not seen with *Aspergillus flavus* (*A. flavus*), a fungus observed through scanning electron microscopy [61].

The surfaces of ABS, HDPE, and PP were smoother and less porous than cardboard. After GS-2 was applied and microorganisms were inoculated, some moisture remained on the surface to allow better interactions of GS-2 with microbial cells. Although ABS, HDPE, and PP appeared to be very similar in smoothness, different GS-2 antimicrobial efficacies were observed. For example, *S. enterica* Agona reductions between different GS-2 concentrations and treatment times were not significantly different on PP coupons (*p* > 0.05, Figure 1D and Figure 3D). A decrease of only ~1.5 log CFU/in^2^ was found for *S. enterica* Agona inoculated on GS-2-treated PP coupons compared to non-GS-2-treated PP coupons, whereas *E. coli* O157:H7 and *L. monocytogenes* populations were reduced by ~6 log CFU/in^2^ on the same PP coupon (*p* < 0.05, Figure 5D). It was not completely clear why GS-2 has a varied efficacy against *S. enterica* Agona on PP coupons, other than the fact that PP coupons are more hydrophobic than polyethylene (PE) coupons and the hydrophobicity might reduce the retention and interaction of GS-2 with *S. enterica* Agona [62]. One possible explanation is that *S. enterica* Agona might have a stronger hydrophobic interaction with PP coupons, allowing better adherence to the surface [62,63]. Follow-up studies using RNA sequencing across all strains on different packaging surfaces could provide additional leads to interpret the variable GS-2 antimicrobial efficacy and elucidate the mode-of-action.

For the virus, treatment with GS-2 for 60 min only achieved a ~1 log reduction in MNV on ABS coupons, whereas on cardboard and PP coupons, GS-2 was not shown to be effective against MNV (Figure 2C). In a previous study, GS-2 (30 mg/mL) with thymol (5 mg/mL) demonstrated broad antiviral efficacy, with 10 min of exposure on glass Petri dishes, which led to a reduction in Herpes Simplex Virus-1 (HSV-1; ATCC/VR-733), poliovirus (ATCC/VR-192), and Murine Hepatitis Virus Strain 1 (MHV-1; ATCC/VR-261) by >6.5 log, 3.0 log, and 5.42 log, respectively [20]. One explanation is that the glass surface is more hydrophilic than the plastic surface [64]. The hydrophilic nature of glass allows better interactions of the GS-2 and MNV and therefore a higher antiviral efficacy compared to plastic. On plastic surfaces, MNV can form a hydrophobic interaction which contributes considerably to MNV adhesion to the surface [63]. The adhesion could potentially shield MNV from exposure to GS-2, contributing to the lower antiviral efficacy of GS-2 on ABS and PP coupons in this study. Additionally, HSV-1 and MHV-1, which were shown to be most susceptible to GS-2 treatment, are both enveloped viruses, while poliovirus and MNV are not. Our work with the enveloped Influenza A virus (Figure S1) also showed significant antiviral activity of GS-2. As mentioned, the antibacterial mechanism of action of capric acid and L-arginine is the induction of membrane permeability. While additional experiments are required to test this hypothesis, the antiviral properties of GS-2 may be a result of envelope disruption.

Some inherent technical limitations should be noted in this study. For instance, we used small 1 in by 1 in plastic and cardboard coupons for the convenience of microbial recovery and enumeration at a laboratory scale, instead of using actual plastic crates or cardboard boxes. In addition, we evaluated the GS-2 efficacy against pure microbial cultures spiked on packaging surfaces at high inoculation levels, which is typical for microbial challenge studies. However, microbial challenge study design cannot represent the lower microbial population levels in an actual event of packaging contamination, nor the potential interference caused by other native microflora (such as non-pathogens) existing on reusable packaging surfaces. These factors should be carefully considered in future validation studies.

## 5. Conclusions

The results from this study demonstrated the antimicrobial efficacy of GS-2 against several bacterial pathogens including *E. coli* O157:H7, *L. monocytogenes*, and *S. enterica* on different reusable food packaging surfaces including cardboard, ABS, HDPE, and PP. The overall stability and antimicrobial effectiveness of GS-2 on reusable packaging shows that this chemical can be a valuable alternative in the efforts to minimize packaging waste while ensuring microbial food safety. We expect that GS-2 spray coating on reusable food packages can benefit American producers and exporters in meeting the increasingly stringent requirements imposed by the EU and other regulatory bodies.

## Data Availability

The original contributions presented in the study are included in the article/supplementary material, further inquiries can be directed to the corresponding author.

## Supplementary Materials

The following supporting information can be downloaded at: https://www.mdpi.com/article/10.3390/foods13213490/s1, Figure S1: Recovery of Influenza A virus (PFU/mL) with no-GS-2 treatment (control) and 3% GS-2 treatment for 0 (GS-2 + 0), 15 (GS-2 + 15), or 60 (GS-2 + 60) minutes on acrylonitrile butadiene styrene (ABS) coupons. Limit of detection is 2 log PFU/mL. Values are presented as mean ± standard deviation. Asterisks (*) represent significant differences between treatments (GS-2 + 0, GS-2 + 15, or GS-2 + 60 vs. control) on coupon.

## References

[1] European Parliament (1994). Directive 94/62/EC on Packaging and Packaging Waste.

[2] European Commission (2018). A European Strategy for Plastics in a Circular Economy.

[3] Van Eygen E., Laner D., Fellner J. (2018). Circular Economy of Plastic Packaging: Current Practice and Perspectives in Austria. Waste Manag..

[4] U.S. Department of Agriculture Global Agricultural Trade System (GATS) Data. https://apps.fas.usda.gov/gats/ExpressQuery1.aspx.

[5] U.S. Centers for Disease Control and Prevention Multistate Foodborne Outbreak Notices. https://www.cdc.gov/foodborne-outbreaks/active-investigations/all-foodborne-outbreak-notices.html.

[6] Economic Research Service U.S. Department of Agriculture Cost Estimates of Foodborne Illnesses. https://www.ers.usda.gov/data-products/cost-estimates-of-foodborne-illnesses.aspx.

[7] Ranjbar A., Ramezanian A., Shekarforoush S., Niakousari M., Eshghi S. (2022). Antifungal Activity of Thymol against the Main Fungi Causing Pomegranate Fruit Rot by Suppressing the Activity of Cell Wall Degrading Enzymes. LWT.

[8] Agrios G.N. (2005). Plant Diseases Caused by Fungi. Plant Pathology.

[9] Olaimat A.N., Taybeh A.O., Al-Nabulsi A., Al-Holy M., Hatmal M.M., Alzyoud J., Aolymat I., Abughoush M.H., Shahbaz H., Alzyoud A. (2024). Common and Potential Emerging Foodborne Viruses: A Comprehensive Review. Life.

[10] O’Shea H., Blacklaws B.A., Collins P.J., McKillen J., Fitzgerald R. (2019). Viruses Associated with Foodborne Infections. Ref. Modul. Life Sci..

[11] Hall A.J., Lopman B.A., Payne D.C., Patel M.M., Gastañaduy P.A., Vinjé J., Parashar U.D. (2013). Norovirus Disease in the United States. Emerg. Infect. Dis..

[12] Burton T.D., Carrera Montoya J., Frota T., Mackenzie J.M. (2024). Human Norovirus Cultivation Models, Immune Response and Vaccine Landscape. Advances in Virus Research.

[13] Iwu C.D., Okoh A.I. (2019). Preharvest Transmission Routes of Fresh Produce Associated Bacterial Pathogens with Outbreak Potentials: A Review. Int. J. Environ. Res. Public Health.

[14] Alegbeleye O.O., Singleton I., Sant’Ana A.S. (2018). Sources and Contamination Routes of Microbial Pathogens to Fresh Produce during Field Cultivation: A Review. Food Microbiol..

[15] Beauchat L.R., Ryu J.-H. (1997). Produce Handling and Processing Practices. Emerg. Infect. Dis..

[16] López-Gálvez F., Rasines L., Conesa E., Gómez P.A., Artés-Hernández F., Aguayo E. (2021). Reusable Plastic Crates (RPCs) for Fresh Produce (Case Study on Cauliflowers): Sustainable Packaging but Potential Salmonella Survival and Risk of Cross-Contamination. Foods.

[17] Ohman E., Kilgore S., Waite-Cusic J., Kovacevic J. (2023). Before and After: Evaluation of Microbial and Organic Loads in Produce Handling and Packing Operations with Diverse Cleaning and Sanitizing Procedures. J. Food Prot..

[18] Ohman E., Waite-Cusic J., Kovacevic J. (2023). Cleaning and Sanitizing in Produce Facilities: Identifying Compliance Gaps and Associated Training Needs, Opportunities and Preferences. Food Prot. Trends.

[19] Simões M., Simões L.C., Vieira M.J. (2010). A Review of Current and Emergent Biofilm Control Strategies. LWT.

[20] Mayfosh A.J., Day Z.I., Unsworth N.B., Liu C.Q., Gupta R., Haynes S., Abraham R., Abraham S., Shaw Z.L., Walia S. (2022). GS-2: A Novel Broad-Spectrum Agent for Environmental Microbial Control. Biomolecules.

[21] U.S. Food & Drug Administration CFR—Code of Federal Regulations Title 21. https://www.accessdata.fda.gov/scripts/cdrh/cfdocs/cfcfr/CFRSearch.cfm?fr=172.785.

[22] U.S. Food & Drug Administration CFR—Code of Federal Regulations Title 21. https://www.accessdata.fda.gov/scripts/cdrh/cfdocs/cfcfr/cfrsearch.cfm?fr=172.860.

[23] U.S. Food & Drug Administration CFR—Code of Federal Regulations Title 21. https://www.accessdata.fda.gov/scripts/cdrh/cfdocs/cfcfr/CFRSearch.cfm?fr=582.5145.

[24] U.S. Food & Drug Administration CFR—Code of Federal Regulations Title 21. https://www.accessdata.fda.gov/scripts/cdrh/cfdocs/cfcfr/CFRSearch.cfm?fr=172.515&SearchTerm=thymol.

[25] Yoon B.K., Jackman J.A., Valle-González E.R., Cho N.J. (2018). Antibacterial Free Fatty Acids and Monoglycerides: Biological Activities, Experimental Testing, and Therapeutic Applications. Int. J. Mol. Sci..

[26] Min D.B., Ellefson W.C., Nielsen S.S. (2010). Food Analysis.

[27] Patra C.N., Sahu K., Singha R., Jena G.K., Jammula S., Das N.R. (2024). Multifaceted Applications of Solid Lipid: A Comprehensive Review. Biomed. Mater. Devices.

[28] McCollum J.T., Cronquist A.B., Silk B.J., Jackson K.A., O’Connor K.A., Cosgrove S., Gossack J.P., Parachini S.S., Jain N.S., Ettestad P. (2013). Multistate Outbreak of Listeriosis Associated with Cantaloupe. N. Engl. J. Med..

[29] Grant J., Wendelboe A.M., Wendel A., Jepson B., Torres P., Smelser C., Rolfs R.T. (2008). Spinach-Associated *Escherichia coli* O157:H7 Outbreak, Utah and New Mexico, 2006. Emerg. Infect. Dis..

[30] Centers for Disease Control and Prevention Multistate Outbreak of Salmonella Serotype Agona Infections Linked to Toasted Oats Cereal—United States, April–May, 1998. https://www.cdc.gov/mmwr/preview/mmwrhtml/00053368.htm.

[31] Hwang S., Alhatlani B., Arias A., Caddy S.L., Christodoulou C., Cunha J., Emmott E., Gonzalez-Hernandez M., Kolawole A., Lu J. (2014). Murine Norovirus: Propagation, Quantification and Genetic Manipulation. Curr. Protoc. Microbiol..

[32] Guimarães A., Venâncio A. (2022). The Potential of Fatty Acids and Their Derivatives as Antifungal Agents: A Review. Toxins.

[33] Liang N., Cai P., Wu D., Pan Y., Curtis J.M., Gänzle M.G. (2017). High-Speed Counter-Current Chromatography (HSCCC) Purification of Antifungal Hydroxy Unsaturated Fatty Acids from Plant-Seed Oil and Lactobacillus Cultures. J. Agric. Food Chem..

[34] Chen Y.Y., Liang N.Y., Curtis J.M., Gänzle M.G. (2016). Characterization of Linoleate 10-Hydratase of Lactobacillus Plantarum and Novel Antifungal Metabolites. Front. Microbiol..

[35] Black B.A., Zannini E., Curtis J.M., Gänzle M.G. (2013). Antifungal Hydroxy Fatty Acids Produced during Sourdough Fermentation: Microbial and Enzymatic Pathways, and Antifungal Activity in Bread. Appl. Environ. Microbiol..

[36] Martin-Arjol I., Bassas-Galia M., Bermudo E., Garcia F., Manresa A. (2010). Identification of Oxylipins with Antifungal Activity by LC-MS/MS from the Supernatant of Pseudomonas 42A2. Chem. Phys. Lipids.

[37] McCarthy M.W., Walsh T.J. (2018). Amino Acid Metabolism and Transport Mechanisms as Potential Antifungal Targets. Int. J. Mol. Sci..

[38] Kim C., Jung H., Kim Y.O., Shin C.S. (2006). Antimicrobial Activities of Amino Acid Derivatives of Monascus Pigments. FEMS Microbiol. Lett..

[39] Tian J., Ban X., Zeng H., He J., Huang B., Wang Y. (2011). Chemical Composition and Antifungal Activity of Essential Oil from *Cicuta Virosa* L. Var. Latisecta Celak. Int. J. Food Microbiol..

[40] Bernardos A., Marina T., Žáček P., Pérez-Esteve É., Martínez-Mañez R., Lhotka M., Kouřimská L., Pulkrábek J., Klouček P. (2015). Antifungal Effect of Essential Oil Components against Aspergillus Niger When Loaded into Silica Mesoporous Supports. J. Sci. Food Agric..

[41] Yoshimi A., Miyazawa K., Abe K. (2016). Cell Wall Structure and Biogenesis in Aspergillus Species. Biosci. Biotechnol. Biochem..

[42] Ahmadipour S., Field R.A., Miller G.J. (2021). Prospects for Anti-Candida Therapy through Targeting the Cell Wall: A Mini-Review. Cell Surf..

[43] Barthel L., Cairns T., Duda S., Müller H., Dobbert B., Jung S., Briesen H., Meyer V. (2024). Breaking down Barriers: Comprehensive Functional Analysis of the Aspergillus Niger Chitin Synthase Repertoire. Fungal Biol. Biotechnol..

[44] Miramón P., Pountain A.W., Lorenz M.C. (2023). Candida Auris-Macrophage Cellular Interactions and Transcriptional Response. Infect. Immun..

[45] Greenway D.L.A., Dyke K.G.H. (1979). Mechanism of the Inhibitory Action of Linoleic Acid on the Growth of Staphylococcus Aureus. J. Gen. Microbiol..

[46] Chamberlain N.R., Mehrtens B.G., Xiong Z., Kapral F.A., Boardman J.L., Rearick4 J.I. (1991). Correlation of Carotenoid Production, Decreased Membrane Fluidity, and Resistance to Oleic Acid Killing in Staphylococcus Aureus 18Z. Infect. Immun..

[47] Westergren I., Johansson B.B. (1993). Altering the Blood-brain Barrier in the Rat by Intracarotid Infusion of Polycations: A Comparison between Protamine, poly-L-lysine and poly-L-arginine. Acta Physiol. Scand..

[48] Nemoto E., Takahashi H., Kobayashi D., Ueda H., Morimoto Y. (2006). Effects of Poly-L-Arginine on the Permeation of Hydrophilic Compounds through Surface Ocular Tissues. Biol. Pharm. Bull..

[49] Li L., Vorobyov I., Allen T.W. (2013). The Different Interactions of Lysine and Arginine Side Chains with Lipid Membranes. J. Phys. Chem. B.

[50] Jalal R., Sepahi M., Mashreghi M. (2017). Antibacterial Activity of Poly-l-Arginine under Different Conditions. Iran. J. Microbiol..

[51] Ram A.F.J., Arentshorst M., Damveld R.A., vanKuyk P.A., Klis F.M., van den Hondel C.A.M.J.J. (2004). The Cell Wall Stress Response in Aspergillus Niger Involves Increased Expression of the Glutamine: Fructose-6-Phosphate Amidotransferase-Encoding Gene (gfaA) and Increased Deposition of Chitin in the Cell Wall. Microbiology.

[52] Hingston P.A., Piercey M.J., Hansen L.T. (2015). Genes Associated with Desiccation and Osmotic Stress in Listeria Monocytogenes as Revealed by Insertional Mutagenesis. Appl. Environ. Microbiol..

[53] Kragh M.L., Truelstrup Hansen L. (2020). Initial Transcriptomic Response and Adaption of Listeria Monocytogenes to Desiccation on Food Grade Stainless Steel. Front. Microbiol..

[54] Welsh D.T., Herbert R.A. (1999). Osmotically Induced Intracellular Trehalose, but Not Glycine Betaine Accumulation Promotes Desiccation Tolerance in *Escherichia coli*. FEMS Microbiol. Lett..

[55] Manzanera M., Vilchez S., Tunnacliffe A. (2004). High Survival and Stability Rates of *Escherichia Coli* Dried in Hydroxyectoine. FEMS Microbiol. Lett..

[56] Maserati A., Fink R.C., Lourenco A., Julius M.L., Diez-Gonzalez F. (2017). General Response of *Salmonella enterica* Serovar Typhimurium to Desiccation: A New Role for the Virulence Factors sopD and sseD in Survival. PLoS ONE.

[57] Suehr Q.J., Chen F., Anderson N.M., Keller S.E. (2020). Effect of pH on Survival of *Escherichia coli* O157, *Escherichia coli* O121, and *Salmonella enterica* During Desiccation and Short-Term Storage. J. Food Prot..

[58] Silhavy T.J., Kahne D., Walker S. (2010). The Bacterial Cell Envelope. Cold Spring Harb. Perspect. Biol..

[59] Kazmierczak M.J., Mithoe S.C., Boor K.J., Wiedmann M. (2003). Listeria Monocytogenes σB Regulates Stress Response and Virulence Functions. J. Bacteriol..

[60] Patrignani F., Siroli L., Gardini F., Lanciotti R. (2016). Contribution of Two Different Packaging Material to Microbial Contamination of Peaches: Implications in Their Microbiological Quality. Front. Microbiol..

[61] Siroli L., Patrignani F., Serrazanetti D.I., Chiavari C., Benevelli M., Grazia L., Lanciotti R. (2017). Survival of Spoilage and Pathogenic Microorganisms on Cardboard and Plastic Packaging Materials. Front. Microbiol..

[62] Hadiyanto H., Khoironi A., Dianratri I., Suherman S., Muhammad F., Vaidyanathan S. (2021). Interactions Between Polyethylene and Polypropylene Microplastics and *Spirulina* sp. Microalgae in Aquatic Systems. Heliyon.

[63] Trudel-Ferland M., Goetz C., Girard M., Curt S., Mafu A.A., Fliss I., Jean J. (2021). Physicochemical Parameters Affecting Norovirus Adhesion to Ready-To-Eat Foods. Appl. Environ. Microbiol..

[64] Song Y., Dunleavy M., Li L. (2023). How to Make Plastic Surfaces Simultaneously Hydrophilic/Oleophobic?. ACS Appl. Mater. Interfaces.

