# Supplementary material for: Antimicrobial Efficacy of GS-2 on Reusable Food Packaging Materials for Specialty Crops
Source: Foods. 2024 Oct 31;13(21):3490. doi: 10.3390/foods13213490 (PMC11545833; doi:10.3390/foods13213490)
Supplement: Supplementary file 1 [file foods-13-03490-s001.zip › foods-3264950-supplementary.pdf]

Supplementary Materials:

GS-2 was highly effective against Influenza A virus as the addition of GS-2 at only 0 min significantly reduced Influenza A virus by ~2.2 log PFU/mL on ABS coupons (*p* < 0.05, Figure S1). After 15 min and 60 min of GS-2 exposure, Influenza A virus was reduced to below the detection limit, resulting in a >3 log PFU/mL reduction (*p* < 0.05, Figure S1).

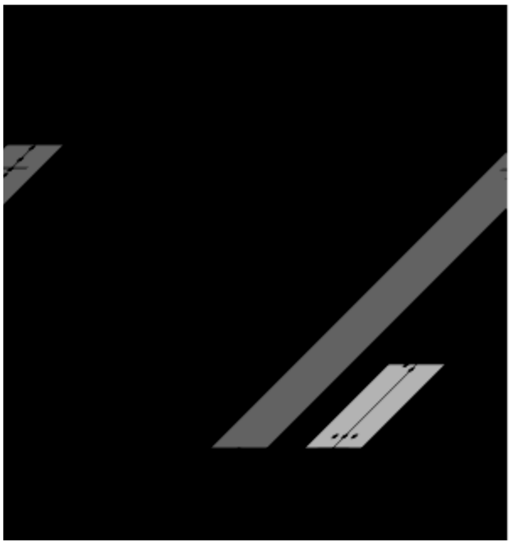

Figure S1: Recovery of Influenza A virus (PFU/mL) with no-GS-2 treatment (control) and 3% GS-2 treatment for 0 (GS-2 + 0), 15 (GS-2 + 15), or 60 (GS-2 + 60) minutes on acrylonitrile butadiene styrene (ABS) coupons. Limit of detection is 2 log PFU/mL. Values are presented as mean ± standard deviation. Asterisks (*) represent significant differences between treatments (GS-2 + 0, GS-2 + 15, or GS-2 + 60 vs. control) on coupon.
